# Integrins, CAFs and Mechanical Forces in the Progression of Cancer

**DOI:** 10.3390/cancers11050721

**Published:** 2019-05-24

**Authors:** Imjoo Jang, Karen A. Beningo

**Affiliations:** Department of Biological Sciences, Wayne State University, Detroit, MI 48202, USA; imjoo@wayne.edu

**Keywords:** integrin, CAF, cancer-associated fibroblast, cancer cells, mechanics of cancer, mechanotransduction, TME, tumor microenvironment, ECM, extracellular matrix, ECM remodeling, ECM proteins

## Abstract

Cells respond to both chemical and mechanical cues present within their microenvironment. Various mechanical signals are detected by and transmitted to the cells through mechanoreceptors. These receptors often contact with the extracellular matrix (ECM), where the external signals are converted into a physiological response. Integrins are well-defined mechanoreceptors that physically connect the actomyosin cytoskeleton to the surrounding matrix and transduce signals. Families of α and β subunits can form a variety of heterodimers that have been implicated in cancer progression and differ among types of cancer. These heterodimers serve as the nexus of communication between the cells and the tumor microenvironment (TME). The TME is dynamic and composed of stromal cells, ECM and associated soluble factors. The most abundant stromal cells within the TME are cancer-associated fibroblasts (CAFs). Accumulating studies implicate CAFs in cancer development and metastasis through their remodeling of the ECM and release of large amounts of ECM proteins and soluble factors. Considering that the communication between cancer cells and CAFs, in large part, takes place through the ECM, the involvement of integrins in the crosstalk is significant. This review discusses the role of integrins, as the primary cell-ECM mechanoreceptors, in cancer progression, highlighting integrin-mediated mechanical communication between cancer cells and CAFs.

## 1. Introduction

The tumor microenvironment (TME) is composed of extracellular matrix (ECM), structural elements of ECM, stromal cells and soluble factors such as growth factors and cytokines [1,2]. The ECM benefits resident cells by providing structural and biochemical support as it consists of fibrous proteins, proteoglycans and multiadhesive glycoproteins [3]. While providing physical support for the neighboring cells, the physical properties of the ECM, including rigidity, elasticity and topology also underwrite many biological processes. Stromal cells in the TME include normal fibroblasts, constitutively activated fibroblasts, immune cells, endothelial cells, smooth muscle cells, pericytes and bone marrow-derived cells. Fibroblasts that are found in the TME, differing from normal fibroblasts, can be identified by expression of certain markers, including α-smooth muscle actin (α-SMA), fibroblast activation protein (FAP), fibroblast-specific protein-1 (FSP1) and platelet derived growth factor receptor α/β (PDGFR α/β). The marker profile of fibroblast depends on the cancer type and quantity and type of excess ECM proteins, such as fibronectin and collagen [4,5]. The perpetually activated fibroblasts in the TME, presenting myofibroblastic markers, are referred to as cancer-associated fibroblasts (CAFs) [4].

Cells respond to diverse types of mechanical stimuli (e.g., substrate rigidity, hydrostatic pressure, compressive, tensile and shear stress) in their microenvironment. These mechanical stimulants can be considered as ligands, similar to biochemical molecules. Upon receipt of these mechanical ligands the cell converts these mechanical cues into biochemical signals through a process known as mechanotransduction [6,7,8,9]. The mechanical information is transduced through mechanoreceptors located between the cell and the ECM or within cell–cell contacts. Mechanotransduction through these receptors effects cellular processes, including cell proliferation, differentiation and migration.

Cellular mechanoreceptors physically interact with the surrounding matrix or other cells within their microenvironments. A number of cell surface receptors are found within the plasma membrane including integrins, syndecans and selectins, that mechanically link the ECM to the intracellular actomyosin cytoskeleton through adhesion molecules. Other surface molecules including cadherins, G-protein coupled receptors, receptor tyrosine kinases, ion channels, lipid rafts and glycocalyx have also been implicated in mechanoreception [10,11,12]. However, among cell surface receptors, integrins are the best described cell-ECM mechanoreceptors and govern cell proliferation, migration, survival, tissue invasion and innate immunity [12]. Integrins function collectively in response to mechanical signals often forming large multiprotein complexes known as focal adhesions. Clusters of integrins connect the cell to the ECM and integrate signals from both sides of the plasma membrane and transduce them bidirectionally [13].

Like normal cells, cancer cells also recognize mechanical changes provided by the TME and convert them into signaling pathways through mechanotransduction. As it is widely accepted that the interplay between cancer cells and their microenvironment, including ECM and stromal cells play crucial roles in cancer progression, evidence is accumulating that cancer cells exploit mechanical signaling pathways [14,15]. CAFs are mechanically competent cells and are the most abundant stromal cell type within the TME [16]. Data suggests that CAFs play a crucial role in cancer development and metastasis [17,18,19,20]. The effects of CAFs in cancer progression result from their secretion of soluble factors such as growth factors, cytokines and ECM molecules. Moreover, the excessive production of fibrillar ECM proteins and ECM remodeling by CAFs in the TME create cancer fibrosis, known as desmoplasia. The CAF-derived increased ECM stiffness not only contributes to CAF differentiation, but also supports cancer progression. As the primary receptors mediating cell-matrix adhesion, integrins expressed by cancer and stromal cells have been implicated in regulating many aspects of cancer progression [13,21]. Here, we discuss the contribution of heterogenous integrins in the mechanics of cancer progression and highlight emerging data describing the crosstalk between cancer cells and the TME, mainly focused on CAFs, mediated through integrins.

## 2. Integrins

Integrins, the best characterized cell-ECM mechanoreceptors, are composed of non-covalently linked heterodimeric α and β subunits. Twenty-four distinct integrin subtypes have been identified in mammals and are made up of eighteen α subunits (α1–11, αV, αIIb, αL, αM, αX, αD and αE) and eight β subunits (β1–8) [11,22]. Each integrin subunit possesses a large extracellular domain, a single transmembrane domain, and a short cytoplasmic tail (except for β4). The α subunits allow integrins to selectively bind to distinct motifs of ECM proteins (Arg-Gly-Asp (RGD) motif, collagen and laminin) [11], whereas the β subunits are responsible for the interaction of integrin with the actomyosin cytoskeleton through numerous anchoring proteins, connecting multiple signaling pathways [23]. Heterodimerization of integrin often takes place within the cell before presentation on the cell surface through the interaction between the β-propeller domain of the α subunit and the hybrid domain of the β subunit [24].

### 2.1. Integrin Activation

Inactive integrin heterodimers are usually found in a bent conformation and possess low affinity for their ligands. Once activated, integrins undergo rapid conformational changes to become extended with increased ligand affinity. Intracellularly, the conformational changes of integrins can be accomplished by binding of intracellular activators (e.g., talin and kindlin) [25,26]. Both activators possess a FERM (4.1/ezrin/radixin/moesin) domain that enables them to bind to the β subunit cytoplasmic tails of integrins. However, whether the activators work together or independently is not yet fully understood. Integrins span the cell membrane and are exposed on both the inside and outside of the cell. Therefore, the activation of integrins can take place in both sides of the membrane, consequently integrins transduce signals bidirectionally across the membrane [27,28]. Integrin activation can occur by biochemical or mechanical ligands. Through their extracellular domains, integrins can be activated by mechanical stimuli from within the ECM and stromal cells, or by binding to ECM proteins, such as collagen, fibronectin, fibrinogen, laminin and vitronectin as their ligands. Integrin clustering is regulated not only by ECM ligands, but by other extracellular integrin interacting partners (e.g., galectins and tetraspanins) as they bind to the extracellular domain of integrins in a ligand-independent manner [21]. Moreover, biochemical and mechanical ligands can be applied reciprocally to activate integrins. In support of this hypothesis, it was found that ligand-induced mechanical force by steered molecular dynamics (SMD) stimulation accelerates hinge-angle opening, resulting in the transition of integrins from an inactive to an active conformation with high ligand-binding affinity [29].

Activated integrins exhibit high affinity to extracellular ligands, leading to integrin clustering at the cell membrane which stabilizes the cell-ECM interaction. Consequently, extracellular signals are transduced through integrins into the cell, known as outside-in signaling that affects various cellular behaviors, including motility, spreading, migration, growth, survival, proliferation and differentiation [30,31]. This signal transduction cannot be solely governed by integrins as they are not capable of phosphorylating or dephosphorylating other molecules in the signaling cascade. Instead, recruited kinases (e.g., FAK, ILK and Src) upon integrin activation carry out the task to deliver the signals to the downstream molecules.

### 2.2. Ligand Specificity and Diversity

Distinct extracellular domains of integrin α subunits give each integrin the binding specificity to proteins of the ECM [24]. For instance, collagen binding integrins consist of α1, α2, α10 or α11 subunits, which heterodimerize only with the β1 subunit, and recognize the same motif, GFOGER. Alternatively, α3, α6, α7 subunits belong to a subgroup of integrins that bind to laminin [11,32]. The number of interacting ECM ligands also varies amongst different integrins, for example, integrin α5β1 recognizes primarily fibronectin, whereas αvβ3 binds to multiple RGD containing proteins such as fibronectin and vitronectin [33]. Even when they interact with the same ligands, the integrins can recognize them in a different manner. For example, although integrins α5β1 and α4β1 are known to bind to fibronectin, their recognition sequences in fibronectin are different. Integrin α5β1 recognizes the RGD sequence in fibronectin, whereas integrin α4β1 recognizes EILDV and REDV sequences.

A different combination of integrin subunits can have a distinct impact on the same cellular response. For instance, in the context of glomerulosclerosis, caused by abnormal collagen deposition in the glomerulus, integrins α1β1 and α2β1 play different roles. Whereas integrin α2β1 binds to collagen type I and positively regulates collagen synthesis, integrin α1β1 interacts with collagen type IV to negatively modulate the synthesis of collagen. As a result, integrin α2β1 exacerbates the condition, but integrin α1β1 protects from glomerular injury, indicating that expression of a subset of integrins can result in contradictory cellular responses based on its ECM ligand [34,35]. Another example of different responses to similar subunit combinations involves αv subunit-containing integrins that bind to the RGD motif. Five different β subunits (β1, β3, β5, β6 and β8) are known to form heterodimers with the αv subunit, but different combinations can give rise to diverse cellular responses [36]. For example, integrin αvβ3, the most studied αv subunit-containing integrin, binds to RGD motif containing proteins (e.g., vitronectin, fibronectin, fibrinogen, and von Willebrand factor), collagen, laminin and also interacts with vascular endothelial growth factor receptor (VEGFR), platelet-derived growth factor (PDGF), fibroblast growth factor-2 (FGF2), matrix metalloproteinase-2 (MMP-2) and insulin [37,38]. Integrin αvβ3 is broadly expressed in different types of cells such as endothelial cells, osteoclasts and blood cells. Notably, the expression of the receptor is high in tumors including activated tumor endothelial cells, glioblastomas, malignant melanomas, breast, lung, pancreatic and prostate carcinomas, but not in resting endothelial cells and normal organ systems [39,40,41,42,43]. Furthermore, integrin αvβ3, in association with vitronectin, has been found to facilitate the differentiation of prostate and breast cancer stem cells (CSCs) and subsequently promote tumor formation [44]. Diverse types of integrins have been implicated in cancer progression (see Table 1).

Integrin αvβ5, another αv subunit-containing integrin, is expressed in glioblastomas together with integrin αvβ3 [45]. In addition, the interaction of integrin αvβ5 with TGFBI (transforming growth factor beta-induced), an ECM interacting protein that contains RGD motif, promotes the activation of FAK signaling pathway, consequently enhancing glycolysis and invasiveness in pancreatic cancer cells [52].

### 2.3. Integrins in Mechanotransduction

Many types of mechanical cues are present in the ECM, including substrate rigidity, fluid shear stress, hydrostatic pressure, tensile and compressive forces, and diverse integrin subtypes are involved in mechanosensing and force transmission.

Tissues exhibit different mechanical properties depending on their locations and functions. For example, brain tissue has an elastic modulus of several hundred pascal whereas that of skeletal muscle is approximately 100 kPa [73], and most solid tumors possess considerably higher stiffness than the surrounding normal tissues [74,75]. The gradual stiffening of the tumor stroma arises from deposition and remodeling of the ECM and is believed to promote cancer progression and metastasis [76,77,78]. Changes in matrix stiffness can be recognized by cells, and more rigid substrates often result in greater cellular forces exerted onto the matrix (traction force) along with increased stress fibers and larger focal adhesions [79]. As a result, the mechanical balance is created by the contractility of the cell and the mechanical properties of the ECM. Various biophysical techniques have been developed to study the diverse mechanical properties of cells in mechanotransduction, including traction force microscopy (TFM), magnetic twisting/pulling cytometry (MTC/MPC), optical tweezers, atomic force microscopy (AFM) and shear flow microfluidic devices [80].

Using TFM and surface plasmon resonance to measure forces exerted by cells and binding dynamics of integrins, it has been determined that binding of breast myoepithelial cells to fibronectin through integrins α5β1 or αvβ6 allows them to sense and adapt to different tissue stiffness. While integrin α5β1 is in charge of responding to the stiffness in healthy tissue, αvβ6 plays the corresponding role in malignant tissue [81]. Our lab has used TFM and mechanosensing experiments to understand the mechanisms by which cells regulate the production of traction force and mechanosensing in response to different types of mechanical stimuli. TFM analysis enabled us to identify calpain 4 as a key regulator in the generation of traction force during migration of embryoninc fibroblasts [82]. Calpain 4 is the small regulatory subunit that forms a heterodimer with one of two large catalytic subunits (calpain 1 and calpain 2) in the calpain family of proteases, and calpains have been implicated in integrin-mediated cell adhesion and migration [83,84,85]. The function of calpain 4 in regulating cellular traction forces was found to be independent of proteolytic activities of catalytic subunits [82]. On the other hand, the two catalytic subunits and the regulatory subunit are necessary for cells to respond to changes in substrate topography and externally applied mechanical stimulation, suggesting distinct regulation of mechanotransduction in the cell in response to different types of mechanical cues. We have also observed an inverse relationship between traction stress and metastatic capacity of breast cancer cells. Traction stress of four murine mammary carcinoma cell lines with varying metastatic capacities were tested, and force production decreased as the metastatic potential and capacity increased [86]. In the same manner, adhesion strength, the number of focal adhesion proteins and the amount of active integrin β1 were also inversely correlated with metastatic ability of cells. It is not surprising that focal adhesion proteins and integrins are closely involved in the force generation and transduction, given that traction forces are generated by the cell and transmitted to the ECM.

The ability of cells to sense stiffness can also be attributed to the integrins. For example, the αvβ3 integrin has been found to recognize the stiffness of fibronectin-coated substrates. Fibroblasts incubated with anti-αvβ3 monoclonal antibody that blocks the interaction between integrin αvβ3 and fibronectin failed to sense the rigidity of fibronectin matrix [87]. Similarly, cells lacking RPTPα, a receptor-like protein tyrosine phosphatase, lost the capability to sense the matrix stiffness. This indicates the function of integrin αvβ3 in association with RPTPα in sensing the rigidity of fibronectin matrix at the leading edge during early spreading.

Force-dependent conformational changes in integrin αLβ2 have also been observed and correlate with dissociation from intercellular adhesion molecule 1 (ICAM-1). Increasing tensile forces, using a biomembrane force probe (BFP), applied on the catch bond between integrin αLβ2 and ICAM-1 lead to a more active conformation with higher affinity for its ligand [88]. Likewise, bending and unbending conformational changes of integrin αvβ3 was found to be dependent on tensile forces [89,90]. Force-dependent binding properties of integrin αvβ3 have been identified by using optical tweezers and force-ramp assays, demonstrating that pulling forces regulate the interaction between integrin αvβ3 and Thy-1 and a slip bond behavior of Thy-1/αvβ3 interaction [91]. As one such approach to understand the influence of tugging forces generated within the tumor stroma on cancer cells, an in vitro mechano-invasion assay was designed to mimic the TME where cancer cells are subjected to mechanical stimuli produced by neighboring highly contractile cells such as CAFs possessing myofibroblastic properties [92]. In this assay, random transient tugging forces are applied to the cells seeded on the collagen type I/fibronectin substrate. The forces on the cancer cells are created by carboxylated paramagnetic microbeads, covalently linked to ECM proteins and randomly positioned, as a rotating magnet twitches the beads surrounding the cells. The applied transient mechanical stimulation led to enhanced invasiveness of highly invasive human fibrosarcoma cells in a fibronectin-dependent manner, but no significant effect was observed in poorly invasive or normal cells. In addition, the pro-invasive effect in response to tugging forces was found to be dependent on cofilin, a known regulator of invadopodia maturation [71]. Forty-six genes were identified as differentially expressed in mechanically stimulated cells, including several downregulated genes. Surprisingly, *ITGB3* (encoding the known mechanoreceptor integrin β3) was found to be downregulated. Integrin β3 binds to fibronectin, a required component needed to increase cell invasion in response to mechanical stimulation [92]. Downregulated integrin β3 expression upon transient mechanical stimulation augments the amount of active cofilin and enhances enzymatic activity of MMP-2 localized in invadopodia, thus promoting the maturation of invadopodia and the invasion of metastatic cells [71].

Integrins also function as mechanoreceptors in response to shear stress. For instance, integrin αvβ3 and integrins containing β1 and β5 subunit can recognize increased shear stress from in vitro flow channels, leading to their associations with the adaptor protein Shc in bovine aortic endothelial cells (BAECs) [93]. Integrin αvβ3 activation by shear stress also leads to enhanced affinity to ECM proteins in BAECs [94]. In further studies, breast cancer cell attachment through interaction with platelets was attributed to the activation status of integrin αvβ3 under blood flow conditions with shear stress [46]. Integrin αvβ3 activation further elicits the metastatic potential in breast cancer cells [46]. Using single-bond BFP and a multiple bond flow chamber, it has been demonstrated that tensile force and shear stress regulate the interaction between integrin α4β1 and its main ligand vascular cell adhesion molecule-1 (VCAM-1) [95].

## 3. Cancer-Associated Fibroblasts (CAFs)

Normal tissue fibroblasts, key regulators of ECM composition and organization, are found in a quiescent state under normal physiological conditions. However, they develop stress fibers in response to mechanical tension generated by their surrounding substrate and thus differentiate into myofibroblasts during wound healing and fibrosis, both conditions requiring tissue remodeling [96,97]. The existence of myofibroblasts was first described during wound healing in granulation tissues [98].

The activated fibroblasts in tumors, CAFs, have similar characteristics of myofibroblasts as both cell types are contractile and express high levels of α-SMA. However, CAFs are more similar to myofibroblasts present in fibrosis than those in wound healing, because myofibroblasts present during wound healing are eventually lost through apoptosis, whereas CAFs and myofibroblasts in fibrosis are perpetually activated [19]. CAFs are highly heterogeneous and they have been found to originate from various cell types in the tumor stroma, including resident fibroblasts, epithelial and endothelial cells via epithelial/endothelial–mesenchymal transition (EMT/EndMT), bone marrow-derived mesenchymal stem cells, adipose tissue-derived stem cells, pericytes and smooth muscle cells [17,99,100]. Their multiple origins may explain why CAFs are heterogenous and have numerous functions in different sites of the body.

### CAF Activation

CAFs become activated by several tumor-derived growth factors, such as transforming growth factor-β (TGFβ), fibroblast growth factor (FGF), platelet-derived growth factors (PDGFs), epidermal growth factors (EGFs), bone morphogenic proteins (BMP) and sonic hedgehog (SHH) as well as cytokines that are present in the surrounding milieu [2,18].

TGF β-triggered anisotropic matrices produced by CAFs stimulate myofibroblastic differentiation of normal fibroblasts, where integrin αvβ5 regulates inhibitory activity of α5β1 against fibroblast differentiation to promote myofibroblastic activation [53]. To be more specific on the mechanism mediated by integrins, normal fibroblasts present active α5β1 on the surface of the cell, but they become activated into CAFs when α5β1 relocates inside the cells. Within desmoplastic ECM, induced αvβ5 signaling promotes the accumulation of intracellular α5β1, thereby promoting myofibroblastic differentiation. Disrupting α3 expression even reversed phenotype of already differentiated CAFs into normal fibroblast-like appearance, thus negatively affecting cell invasion. The expression of integrin αvβ6 by cancer cells can also regulate CAF activation. Co-culturing normal colonic fibroblasts with colorectal cancer cells (CRCs) has identified that the expression levels between CAF markers (α-SMA and FAP) from the fibroblasts and integrin β6 from CRCs are proportionally related [54]. Further, integrin β6 expressed by CRCs was found to regulate CAF activation through active TGF-β, and activated CAFs mediated by integrin β6 were able to promote CRC cell invasion.

CAF activation can also be modulated through epigenetic changes in the cells by long-term exposure with growth factors and cytokines. Epigenetic modification of fibroblasts can give rise to the perpetuation of fibroblast activation, resulting in the failure of them to return to their resting state. One such example is fibroblast activation mediated by TGFβ stimulation. When primary mouse kidney fibroblasts were exposed to TGFβ for a prolonged period of time, hypermethylation of *RASAL1*, encoding a Ras inactivator, catalyzed by DNA methyltransferase Dnmt1 was induced, causing sustained fibroblast activation in renal fibrogenesis [101]. Similarly, long-term exposure to the pro-inflammatory LIF (leukemia inhibitory factor) cytokine can also activate CAFs through epigenetic modifications. LIF stimulation induces STAT3 acetylation that, in turn, causes an epigenetic-dependent loss of expression of the SHP-1 tyrosine phosphatase by Dnmt1 [102]. As a result, the JAK1/STAT3 signaling pathway is constitutively activated through down-regulation of SHP-1 expression, resulting in contractile and pro-invasive effects of CAF.

Increased tumor stroma stiffness attributed to the excessive accumulation of fibrillar ECM proteins produced by CAFs leads to a chronic wound healing-like response toward cancer cells, a condition known as desmoplasia. The highly dense stroma can be found in invasive cancers such as breast, lung, prostate, pancreas, gastrointestinal tract and squamous cell carcinomas and is associated with poor clinical outcomes [18,103,104]. Desmoplasia, together with the uncontrolled proliferation of cancer cells, creates compressive forces within tumors. These compressive forces can also contribute to CAF activation. Normal pancreatic fibroblasts exhibit increased expression of CAF markers, such as α-SMA and periostin, in response to a defined compressive stress produced by an in vitro transmembrane pressure device [105]. Co-culturing with pancreatic cancer cells, compression-induced activated fibroblasts were able to promote migration of cancer cells resembling CAFs. The compressive stress not only regulated CAF activation but also directly affected cancer progression. It has been reported that compressive stress is a crucial factor to determine the shape and growth of tumor spheroids, which is accompanied by concurrent inhibition of cell proliferation and stimulation of apoptosis [106,107,108]. Furthermore, defined compressive stress applied to breast cancer cells induced coordinated migration of breast cancer cells [109].

## 4. Communication Between CAFs and Cancer Cells Mediated by Integrins

### 4.1. ECM Remodeling by CAFs

**Figure 1:**
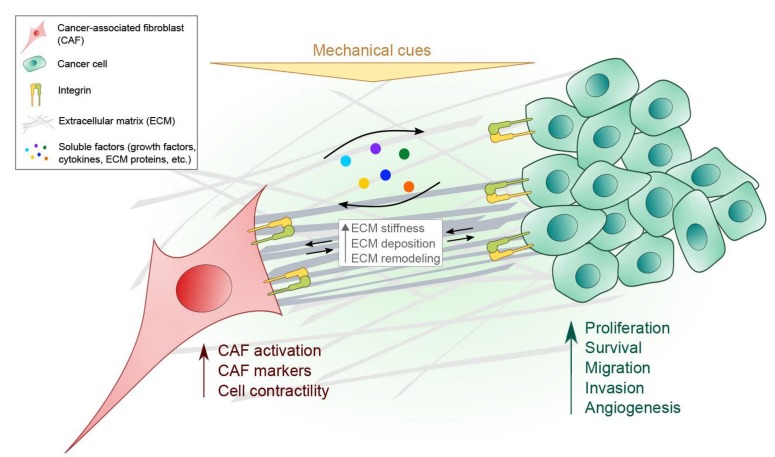
Interplay between CAFs and cancer cells in the TME. Multiple types of integrins (shown as one type for simplification in the figure) on CAFs and cancer cells are responsible for sensing and transducing various protein such as ECM proteins and mechanical cues (e.g., substrate rigidity, hydrostatic pressure, compressive, tensile and shear stress) present in the TME. See Table 1 for details regarding different integrin subtypes and their ligands in cancer progression. Signals arising from enhanced ECM stiffness, for example, can also be recognized by both CAFs and cancer cells through integrins. CAFs and cancer cells also influence each other’s physiological processes by releasing and receiving diverse soluble factors in a paracrine manner.

In the TME, CAFs and cancer cells communicate through the ECM and other soluble factors thereby affecting each other’s cellular behavior (Figure 1). Although this communication is bi-directional, as illustrated in the figure, we have primarily discussed the communication from the CAF to the cancer cell. ECM remodeling by CAFs creates a favorable stromal environment for cancer cells to invade during tumor progression, and the deregulated ECM proteins promote the metastatic cascade. ECM components of the TME, such as collagens, fibronectin and laminins have been widely studied as key regulators in cancer progression [110,111,112]. ECM secretion and remodeling by CAFs have identified the highly expressed integral membrane protein caveolin-1 (Cav1) as facilitating local cancer invasion and metastasis by regulating cell contractility and remodeling via p190RhoGAP [113]. This is consistent with findings that Rho-dependent force-mediated ECM remodeling by CAFs are required to promote cancer cell invasion [56]. Further, integrin α3 and α5 subunits are required in matrix remodeling by CAFs that generate tracks in the matrix, providing guidance for collective invasion of the squamous cell carcinoma (SCC) cells.

Moreover, the significance of collagen type I expression by CAFs has been recognized in tumor initiation, self-renewal potential, cell migration and invasion of pancreatic cancer cells by integrin β1/FAK-dependent signaling pathway [67,68]. Differential gene expression of integrin α11, a fibrillar collagen receptor, in CAFs compared with that of normal fibroblasts has been confirmed in the tumor stroma of non-small cell lung carcinoma (NSCLC). Tumorigenicity of NSCLC was also enhanced when co-implanted with mouse embryonic fibroblasts (MEFs) expressing α11 [62,63,64]. In accordance with these observations, more recent data demonstrated that stromal integrin α11β1, which is highly expressed in CAFs together with α-SMA, induces collagen reorganization and tissue stiffness, promoting tumor growth and metastatic potential in NSCLC [65]. The role of integrin α11 in pancreatic cancer has also been investigated. Knock-down of integrin α11 gene expression in pancreatic stellate cells (PSCs), which are considered precursor cells of CAFs, resulted in lower migration and invasion of tumor cells [66].

Within the stroma, elevated levels of fibronectin expression in CAFs has been identified as a key factor to promote cancer cell migration. The observation of overexpressed fibronectin in tumor stroma has revealed the importance of fibrillar fibronectin-rich matrices produced by head and neck squamous cell carcinomas (HNSCC)-associated fibroblasts [55]. These cells promoted directional migration of collective carcinoma cells mediated through integrins αvβ6 and α9β1 engagement. CAF-derived matrices exhibit more parallel fibronectin organization than that of normal fibroblast. The matrix organization by CAFs is mediated by PDGFRα and myosin-II-driven traction force transduced by integrin α5β1 [60]. As a consequence, the anisotropic alignment of matrix fibers by CAFs enhanced directional pancreatic cancer cell migration by integrin αv. Consistent with these results, integrin αvβ3-regulated fibronectin assembly by CAFs is critical for stimulating colon cancer cell invasion [47]. The changes in matrix organization by CAFs could be due in part to the altered expression levels of CAF markers in tumor stroma. For instance, overexpressing FAP in normal fibroblasts has exhibited aligned fibronectin organization similar to CAF-derived matrix and, subsequently, pancreatic and breast cancer cell invasion was promoted via the activation of integrin β1/FAK signaling pathway [69]. Moreover, fibronectin presented on the surface of CAFs allows cancer cells to interact with CAFs in an integrin-dependent manner. A recent study has determined that direct binding of cancer cells to fibroblasts in the 3D collagen matrix is mediated through the interaction between integrin α5β1 of cancer cells and fibronectin expressed on the surface of fibroblasts [61].

Laminin-322, one of the laminin family of heterotrimers that are major components of the basement membrane, is found to be expressed by CAFs and deposited within the tumor stroma, and its interaction with integrin α3β1 on CAFs is vital for TGFβ-induced CAF differentiation and pancreatic cancer cell invasion [57]. Consistently, higher expression levels of Integrin α3 were observed in pancreatic cancers compared to normal pancreas. Ablation of Integrin α3 expression inhibited tumor growth through blockade of epidermal growth factor receptor (EGFR) signaling pathway accompanied by altered leucine-rich repeats and immunoglobulin-like domain protein 1 (LRIG1) expression. Further, pancreatic cancer patients with high expression of Integrin α3 had lower median survival than the comparable low expressing group [58]. Moreover, a recent study with blocking antibodies against integrin β1 or α3 in vitro revealed integrin α3β1 disruption of cell aggregation and aggregate coalescence in breast cancer, melanoma and glioblastoma cells [59]. Thus, CAF-regulated ECM remodeling in the TME enables CAFs and cancer cells to communicate with each other by exchanging signals that are mainly mediated through integrins, consequently supporting cancer progression.

### 4.2. Paracrine Signaling by CAFs to Cancer Cells

Paracrine signaling through soluble factors mediates communication between CAFs and cancer cells in a bi-directional manner to regulate tumor progression. CAFs become activated in response to soluble factors secreted from cancer cells, and, simultaneously, they express growth factors and cytokines in tumor stroma, thereby contributing to pathways facilitating tumorigenesis and metastasis [20]. For example, stromal IL-6 secreted by CAFs activates epithelial-to-mesenchymal transition (EMT) in esophageal adenocarcinoma (EAC). EAC patient-derived CAFs contribute chemoradiotherapy resistance to EAC cells, and inhibition of IL-6 brings about re-sensitization of tumor cells to therapy [114]. The pro-tumorigenic effects of IL-6 secretion by CAFs have also been demonstrated in other cancer types, such as luminal breast, endometrial and gastric cancers [115,116,117]. A recent finding has revealed that IL-32, which contains the RGD motif, is abundantly secreted by CAFs [72]. By binding to integrin β3 via its RGD motif, the CAF-derived IL-32 stimulates the p38 MAPK signaling pathway, consequently increasing the EMT biomarker expression levels and enhancing breast cancer cell invasion and metastasis.

CAFs also express diverse ECM proteins that regulate tumorigenesis and metastasis. It has been determined that Lumican, an ECM proteoglycan belonging to the family of small leucine-rich repeat proteoglycans (SLRPs), is abundantly expressed by CAFs in gastric cancer (GC) stroma and that Lumican overexpression promotes GC tumorigenesis and metastasis by means of integrin β1/FAK signaling pathway activation [70].

Non-structural ECM proteins known as matricellular proteins, such as tenascin C and periostin are also expressed by CAFs, and their effects on cancer development has widely been reported. Tenascin C, a glycoprotein of the ECM, is highly expressed by CAFs and most malignant solid tumors and affects cancer cell migration and metastasis, which leads to poor clinical outcome in cancer patients. This is evidenced by the finding that tenascin C causes proliferation of human glioblastoma and breast carcinoma cells [118]. Binding of tenascin C to fibronectin weakens cell adhesion as it interferes with syndecan-4 and integrin α5β1 interactions to fibronectin, resulting in enhanced tumor cell proliferation. Tenascin-C expression can also be induced by mechanical stress, and it has been associated with multiple integrin subtypes, including α2β1, α8β1 and α9β1 and αvβ6, although details about how these interactions affect cellular responses remain to be investigated [119]. Another example of CAF-released matricelluar protein promoting tumorigenesis is periostin. Studies have identified periostin as a ligand for integrins αvβ3 and αvβ5 that mediate signaling pathways to enhance cell survival, growth, proliferation, migration, invasion and angiogenesis in various cancer types such as oral, ovarian, breast and colon cancers [48,49,50,51]. Although overexpression of periostin and its importance in cancer progression was commonly observed in various cancers, periostin seems to interact with different sets of integrins [48,49,50]. While periostin functions as a ligand for both integrins αvβ3 and αvβ5 to promote adhesion and migration of ovarian cancer cells, it interacts only with integrin αvβ3 to facilitate metastatic growth and angiogenesis of breast and colon cancer cells. On the other hand, human embryonic kidney cells overexpressing periostin induce cell migration and invasion through interactions with integrin αvβ5, stimulating EGFR signaling pathways and increased proteolytic activity of MMP-9 [120]. The secretion of periostin by CAFs triggers paracrine signaling through which CAF-derived periostin acts as a ligand to bind to integrins expressed on the surface of esophageal cancer cells [121]. Interaction of periostin with integrins αvβ3 and αvβ5 activates PI3K–Akt signaling pathway, resulting in increased tumor cell invasion. The two described matricellular proteins, tenascin C and periostin, expressed by CAFs may mutually contribute to tumor progression as evidenced by the finding revealing that periostin binds to tenascin C and functions as a bridge between tenascin C and the ECM, leading to the incorporation of tenascin-C into the ECM fibrils [122,123].

CAFs also release high levels of urokinase plasminogen activator (u-PA), a serine protease that are found in tumor stroma. Esophageal squamous cell carcinoma (ESCC)-derived CAFs have revealed up-regulated expression of u-PA. The increased u-PA promotes ESCC cell migration and invasion through PI3K/AKT and ERK signaling pathways [124]. Consistent with these observations, increased expression levels and activation of urokinase plasminogen activator (u-PA) and its receptor u-PAR were observed in CAFs purified from patients with symptomatic multiple myeloma, thereby promoting proliferation and invasiveness of human myeloma cells [125]. Podoplanin, a mucin-type sialoglycoprotein, is another known CAF marker. A subsequent number of studies have revealed increased expression of podoplanin by CAFs and its association with tumor progression, metastasis in various cancers, such as breast, lung, colorectal and esophageal cancers [126,127,128,129,130,131]. However, the function of CAF-expressed podoplanin in cancer progression remains unclear, because the overexpression of podoplanin reveals as a negative prognostic factor in some cancers but can have a protective role in others.

## 5. Conclusions

The microenvironment surrounding cancer cells has been recognized as an attractive target for the treatment of cancer patients in the past couple of decades. As the most abundant stromal cells in the TME, CAFs have widely been investigated that they regulate many aspects of tumorigenesis and metastasis. The crosstalk between CAFs and cancer cells takes place either through interchangeable soluble factors in a paracrine manner or through biochemical or mechanical cues within the TME, and the signals are received by their own cell surface receptors. As we discussed in this review, integrins are heterogenous and can form a variety of heterodimers. Various integrins are expressed by different types of cells including CAFs and cancer cells in order to transduce varied signals bidirectionally across the cell membrane. Although some integrins can function in multiple types of cells, the expression or activation of integrins can also be differentially regulated among distinct cell types (e.g., cancer cells vs. normal cells or CAFs vs. normal fibroblasts), therefore, this uniqueness of the receptor would bring about the development of more targeted therapeutic agents.

## Figures and Tables

**Table 1:** Integrins in cancer progression.

Integrins	Source of Expression	Ligands in the Study	Cancer-Promoting Effects	References
αvβ3	Prostate and breast CSC	Vitronectin	Tumor formation	[44]
	Glioblastoma		Glioma-associated angiogenesis	[45]
	Breast cancer	Shear stress	Metastatic potential	[46]
	CAF	Fibronectin	Colon cancer cell invasion	[47]
	CAF	Periostin	Cell survival, growth, proliferation, migration, invasion and angiogenesis	[48,49,50,51]
αvβ5	Glioblastoma		Glioma-associated angiogenesis	[45]
	Pancreatic cancer	TGFBI	Pancreatic cancer cell invasion	[52]
	CAF		Differentiation of normal fibroblasts into CAFs	[53]
	CAF	Periostin	Cell survival, growth, proliferation, migration, invasion and angiogenesis	[48,49,50,51]
αvβ6	CRC		CAF activation and CRC invasion	[54]
	HNSCC	Fibronectin	Directional migration of collective HNSCC cells	[55]
α3β1	CAF		Collective invasion of SCC cells	[56]
	CAF	Laminin-322	CAF differentiation	[57]
	Pancreatic cancer		Poor clinical outcome in pancreatic cancer patients	[58]
	Breast cancer, melanoma and glioblastoma		Cell aggregation and aggregate coalescence	[59]
α5β1	CAF	Fibronectin	Differentiation of normal fibroblasts into CAFs	[53]
	CAF		Collective invasion of SCC cells	[56]
	CAF	Fibronectin	Integrin αv-mediated directional pancreatic cancer cell migration	[60]
	Pancreatic and lung cancer	Fibronectin	Direct interaction of cancer cells with fibronectin-presenting fibroblasts	[61]
α9β1	HNSCC	Fibronectin	Directional migration of collective HNSCC cells	[55]
α11β1	CAF	Collagen	Non-small cell lung carcinoma progression	[62,63,64,65]
	PSC	Collagen	Pancreatic cancer cell migration and invasion	[66]
β1	Pancreatic cancer	Collagen	Tumor initiation, self-renewal potential, migration and invasion	[67,68]
	Pancreatic and breast cancer	Fibronectin	Pancreatic and breast cancer cell invasion	[69]
	Gastric cancer		Tumorigenesis and metastasis of gastric cancer	[70]
β3	Fibrosarcoma	Transient tugging and pulling forces	Integrin β3 downregulation-mediated fibrosarcoma cell invasion	[71]
	Breast cancer	IL-32	Breast cancer cell invasion and metastasis	[72]
